# From advocacy to action: Projecting the health impacts of climate change

**DOI:** 10.1371/journal.pmed.1002624

**Published:** 2018-07-31

**Authors:** Hannah Nissan, Declan Conway

**Affiliations:** 1 International Research Institute for Climate and Society, Columbia University, New York, New York, United States of America; 2 Grantham Research Institute on Climate Change and the Environment, London School of Economics and Political Science, London, United Kingdom

## Abstract

In a Perspective, Hannah Nissan and Declan Conway discuss the implications of uncertainty about projected impacts of climate change on health.

Mitigating climate change by reducing greenhouse gas emissions has many health co-benefits and is a top public health priority. Policies to limit emissions are associated with improvements across a wide range of public health outcomes, including, among other impacts, obesity, acute respiratory infections among children, and ischaemic heart disease in adults [1]. However, recognition that climate change is already underway has led to an increasing focus on adaptation. Studies projecting the impacts of future climate change on health date back to the late 1980s, and their number has grown substantially in recent years. Climate change impact assessments generally use the output of global climate models (GCMs). Here, we profile, and suggest means for addressing, the challenges associated with the use of GCM projections for impact studies to inform adaptation.

GCMs provide projections of the climate at a typical resolution of about 100 km^2^. Such low precision is of limited use to decision-makers trying to determine how climate change might affect their particular district, town, or even country. Often, a regional climate model is employed to ‘downscale’ the output of the global model to a resolution considered more useful for practical applications. Climate model output can then be used to drive disease models or to investigate the risks of surpassing health-relevant climate thresholds in the future.

The outputs of these analyses are often explicitly intended to inform the development of adaptation strategies and plans. However, there is little evidence that climate change projections are used to inform practical adaptation decisions [2–4]. Climate change projections typically target the future several decades ahead, or even at the end of the century. Yet the most pressing issues faced by people and institutions often necessitate a focus on the present and near-term future, particularly in developing countries where there is less capacity to act [3]. Heat waves, for example, are an increasing risk to human health in a warming climate, but many of the most effective strategies to reduce this risk concern the development of seasonal adaptation plans and early warning systems [5]. These programs allow a range of preparedness measures to reduce vulnerability and exposure, from training and awareness-raising activities at the start of the summer season to emergency interventions that could include public alerts, opening cooling centres, and distributing drinking water. In India, for example, moving the neonatal ward from the top to the bottom floor of a hospital had a significant protective effect and required no forecast information at all [6]. Even when the long term is relevant, as, for example, in the case of large infrastructure developments, the uncertainties involved in climate prediction on local scales are so large that they can exceed the magnitude of the projected change [7]. Moreover, with many relevant non-climate factors, it is difficult to disentangle the role that climate change projections may have played in the development of an adaptation plan.

## What we can and cannot say about the future climate

Uncertainty in climate change prediction arises from multiple sources: (1) an imperfect ability to measure and initialize simulations with the current state of the climate system, atmospheric greenhouse gas, and aerosol concentrations (‘initial conditions’); (2) uncertainty about the anticipated future trajectory of greenhouse gas and aerosol emissions; (3) climate model errors leading to uncertainty about how the climate system will respond to this external forcing; and (4) natural climate variability. To quantify the likelihood of different climate futures, multiple model simulations are run, which attempt to sample the range of prediction uncertainty arising from these different sources. These simulations assume different emissions trajectories, use a range of climate models, and are initialized using slightly perturbed initial conditions to see how each of these factors contributes to the total uncertainty. The spread of projected outcomes is taken as an indication of the uncertainty, and probabilities are assigned to outcomes according to how frequently they occur within the ensemble. The problem is that it is impossible to sample the full range of uncertainty within such an ensemble of projections [8,9]. Differences in projections among models are examined closely, but the ensemble of available models is ad hoc and cannot be expected to provide a reliable estimate of the range of futures that might plausibly occur. Moreover, without past test cases over which to calibrate the ensemble projections, it is impossible to know whether probabilistic climate change projections are reliable [8].

These limitations pertain to projections of future climate change at any scale. Obtaining information at local scales and at specific points in the future gives rise to a number of additional issues, which are often overlooked in studies projecting future health impacts. Scientists have high confidence in several aspects of large-scale climate change, including, for example, global warming and large-scale temperature trends and sea level rise. However, the models have many documented limitations, particularly regarding their ability to capture extremes, which are often of most interest for impacts [10]. Projections among models can differ dramatically, especially on scales smaller than continents and even for the direction of change in rainfall in many parts of the world [11]. Downscaled climate information may appear to be a solution, as the output of this process delivers information that appears more realistic because of its higher resolution. However, regional downscaling cannot rectify many of the problems with global models and can give a false impression of confidence [12].

Of all the challenges associated with predicting climate change impacts, the natural variability of the climate system is perhaps the most overlooked. Unlike weather or seasonal forecasts, which are initialized with current weather and climate observations, climate change projections are uninitialized. The models are able to reproduce key modes of natural climate variability on interannual and decadal timescales, but without initialization, the timing of these cycles does not coincide with the real world. Initialised decadal predictions offer promise, but they are currently experimental and do not perform well enough to inform decision-making directly, particularly on local scales and for precipitation [13]. Interannual fluctuations are, overwhelmingly, the largest contributor to total climate variability for both rainfall and temperature. Decadal variability can be significant as well. For example, East Africa has experienced a decline in rainfall since the late 1990s despite long-term projections suggesting that the region is heading for wetter conditions by the end of the century [14]. The ‘global warming hiatus’, when upward temperature trends stalled at the beginning this century, is another example [15]. The science behind global warming is unequivocal, but the expectation that the temperature will be hotter at the end of the century says nothing about the trajectory between now and the long-term future. Failure to consider these fluctuations could have major consequences for adaptation planning, particularly when looking at the next 10 to 30 years [16].

Presentations of future impacts require explicit communication of uncertainties, which includes realistic levels of precision [17] and clear guidance on the relevance of the information (or not) for planning and decision-making. The process of delineating their limitations may, in itself, be enough to deter decision-makers from direct use. Climate model outputs cannot be used to infer local conditions, and they perform especially poorly at the level of an individual model grid. Nor can we use climate projections to infer anything about the future climate over periods shorter than 30 years. Because projections do not capture the timing of interannual and decadal variations, statistics should always be calculated over at least three decades. Extracting model output over shorter windows of time could result in a substantial over- or underestimation of the trend, particularly over the next 10 to 30 years. Alternatively, the output from several models can be averaged to cancel out the different phases of variability in each model. However, only the trend remains after this multi-model averaging is performed; interannual to decadal variability is an additional source of uncertainty in the projections that should be factored into future scenarios, for example, by taking past variability as an indicator of variability in the future [18,19]. Finally, we cannot set too much store by probabilistic projections because the ensemble of models used in the projections is not an accurate representation of the full range of possible futures [9]. The complexities of these considerations point to the importance of close collaboration between climate and health experts when conducting research on future impacts. Failure to capture the full range of uncertainty in decisions could lead to maladaptation [20].

## Long-term impacts, short-term actions

If long-term climate prediction is so uncertain, where is the value in modelling the health impacts of future climate change? Long-term projections are one of many lines of evidence that help to shape climate and health policy by their gradual influence on the culture and priorities of people and institutions. Research on the health impacts of future climate change thus plays an important role in the climate change discourse, but its value is primarily in shaping policy by providing material that can be used to advocate for both mitigation and adaptation programming rather than triggering practical actions. Much of the published information on climate change health impacts serves this advocacy agenda (e.g., WHO’s Climate and Health Country Profile Project [21]). The language used to promote such materials, however, often suggests that they are intended to guide practical adaptation decisions. The high precision of the information that is generally provided gives the misleading impression of high confidence in very specific outcomes.

Practical adaptation measures need to focus on what can be accomplished today with available and reliable climate information while keeping the long term in mind. For example, warming in Ethiopia is raising the maximum elevation for malaria transmission in mountainous areas, exposing new highland populations to malaria risk, but projected temperature trends are uncertain [22]. A suitable adaptation response might use seasonal forecasts to advocate for new surveillance and clinics in marginal transmission zones, with higher vigilance during El Niño years when climate anomalies are more predictable [23] and highland warming is often strongest [22]. Such windows of enhanced predictability can be used to push for malaria eradication, a priority of the WHO Strategic Advisory Group on Malaria Eradication, by putting additional resources into control programs like bednet distribution, indoor residual spraying, and vector control at these times [24]. Approaches to decision-making under uncertainty are attracting attention and provide some promise for planners to incorporate uncertain future climate projections into planning decisions. Rather than a “predict then act” approach, they assess risks to policies [25]. These methods require deep consultation with stakeholders, considerable technical capacity, financial resources, and experience of facilitation. As yet, there are few practical examples in high-income countries and even fewer in low-income countries [26] although examples are now emerging, such as the implementation of a long-term water resources master plan in Lima, Peru [27]. Whilst flexibility can sometimes be built into long-term decisions—even in the case of sunk infrastructure projects [28]—more gradual adaptation options are also available, such as decisions to invest in monitoring and surveillance, reducing vulnerabilities, research, and capacity building [24,29,30].

The modelling of future health impacts has an important role to play in motivating these types of adaptation decisions so that the systems and expertise needed to manage changing climate-related health risks are in place. Studies that project the health impacts of climate change should avoid overselling the utility of this information for practical adaptation by clearly presenting uncertainties and being realistic about the value of projections for shaping policy, rather than triggering actions.

## Abbreviation

GCM: global climate model

## References

[1] Haines A, McMichael AJ, Smith KR, Roberts I, Woodcock J, Markandya A, et al Health and Climate Change 6: Public health benefits of strategies to reduce greenhouse-gas emissions: overview and implications for policy makers. Lancet. 2009;374:2104–14. 10.1016/S0140-6736(09)61759-1 19942281

[2] Kiem AS, Austin EK. Disconnect between science and end-users as a barrier to climate change adaptation. Clim Res. 2013;58(1):29–41.

[3] Jones L, Dougill A, Jones RG, Steynor A, Watkiss P, Kane C, et al Ensuring climate information guides long-term development A shortfall in knowledge and data. Nat Clim Chang. 2015;5:812–4.

[4] Berrang-Ford L, Ford JD, Paterson J. Are we adapting to climate change? Glob Environ Chang. 2011;21(1):25–33.

[5] Knowlton K, Kulkarni SP, Azhar GS, Mavalankar D, Jaiswal A, Connolly M, et al Development and implementation of South Asia’s first heat-health action plan in Ahmedabad (Gujarat, India). Int J Environ Res Public Health. 2014;11(4):3473–92. 10.3390/ijerph110403473 24670386 PMC4024996

[6] Kakkad K, Barzaga ML, Wallenstein S, Azhar GS, Sheffield PE. Neonates in Ahmedabad, India, during the 2010 heat wave: a climate change adaptation study. J Environ Public Health. 2014; 2014:946875 10.1155/2014/946875 24734050 PMC3964840

[7] Charlton MB, Arnell NW. Adapting to climate change impacts on water resources in England—An assessment of draft Water Resources Management Plans. Glob Environ Chang. 2011;21(1):238–48.

[8] Tebaldi C, Knutti R. The use of the multi-model ensemble in probabilistic climate projections. Philos Trans A Math Phys Eng Sci. 2007;365(1857):2053–75. 10.1098/rsta.2007.2076 17569654

[9] Stainforth DA, Allen MR, Tredger ER, Smith LA. Confidence, uncertainty and decision-support relevance in climate predictions. Philos Trans A Math Phys Eng Sci. 2007;365(1857):2145–61. 10.1098/rsta.2007.2074 17569656

[10] Sillmann J, Kharin VV, Zhang X, Zwiers FW, Bronaugh D. Climate extremes indices in the CMIP5 multimodel ensemble: Part 1. Model evaluation in the present climate. J Geophys Res Atmos. 2013;118(4):1716–33.

[11] IPCC. Stocker TF, Qin D, Plattner GK, Tignor M, Allen SK, Boschung A, Nauels A, Xia Y, Bex V, Midgley PM (Eds). Climate Change 2013 Physical Science Basis. Contribution of Working Group I to the Fifth Assessment Report of the Intergovernmental Panel on Climate Change. Cambridge, UK, and New York, New York, USA: Cambridge University Press; 2013:1029–1136. 10.1017/CB09781107415324

[12] Trzaska S, Schnarr E. A review of downscaling methods for climate change projections. United States Agency Int Dev by Tetra Tech ARD. 2014; http://www.ciesin.org/documents/Downscaling_CLEARED_000.pdf

[13] Goddard L, Kumar A, Solomon A, Smith D, Boer G, Gonzalez P, et al A verification framework for interannual-to-decadal predictions experiments. Clim Dyn. 2013;40(1–2):245–72.

[14] Lyon B, Vigaud N. Unraveling East Africa’s Climate Paradox In: Wang S-YS, Yoon J-H, Funk CC, Gillies RR, editors. Climate Extremes: Patterns and Mechanisms. John Wiley & Sons, Inc.; 2017 p. 265–81.

[15] Kosaka Y, Xie S-P. Recent global-warming hiatus tied to equatorial Pacific surface cooling. Nature. 2013;501(7467):403–7. 10.1038/nature12534 23995690

[16] Conway D. Adapting climate research for development in Africa. Wiley Interdiscip Rev Clim Chang. 2011;2(3):428–50.

[17] Hassenzahl DM. Implications of Excessive Precision for Risk Comparisons: Lessons from the Past Four Decades. Risk Anal. 2006;26(1):265–76. 10.1111/j.1539-6924.2006.00719.x 16492197

[18] Greene AM, Goddard L, Gonzalez PLM, Ines AVM, Chryssanthacopoulos J. A climate generator for agricultural planning in southeastern South America. Agric For Meteorol. 2015;203:217–28.

[19] Greene AM, Hellmuth M, Lumsden T. Stochastic decadal climate simulations for the Berg and Breede Water Management Areas, Western Cape province, South Africa. Water Resour Res. 2012;48(6). 10.1029/2011WR011152

[20] Hall J. Probabilistic climate scenarios may misrepresent uncertainty and lead to bad adaptation decisions. Hydrol Process. 2007;21(8):1127–9.

[21] WHO, UNFCCC. Climate and Health Country Profile Project [Internet]. World Health Organisation. Available from: http://www.who.int/globalchange/resources/countries/en/. [cited 25 Jun 2018].

[22] Lyon B, Dinku T, Raman A, Thomson MC. Temperature suitability for malaria climbing the Ethiopian Highlands. Environ Res Lett. 2017;12(064015). 10.1088/1748-9326/aa64e6

[23] Goddard L, Dilley M, Goddard L, Dilley M. El Niño: Catastrophe or Opportunity. J Clim. 2005 3;18(5):651–65.

[24] Nissan H, Ukawuba I, Thomson M. Factoring climate change into the malaria eradication strategy of the World Health Organisation. Report for the Strategic Advisory Group on Malaria Eradication. 2017.

[25] Lempert RJ, Groves DG, Popper SW, Bankes SC. A General, Analytic Method for Generating Robust Strategies and Narrative Scenarios. Manage Sci. 2006;52(4):514–28.

[26] Bhave AG, Dessai S, Stainforth DA. Barriers and opportunities for robust decision making approaches to support climate change adaptation in the developing world. Clim Risk Manag. 2016;14 10.1016/j.crm.2016.09.004

[27] Kalra N, Groves DG, Bonzanigo L, Molina E, Cayo P, Carter R, et al. Robust Decision-Making in the Water Sector. A Strategy for Implementing Lima’s Long-Term Water Resources Master Plan. World Bank Group; 2015. Report No.: 7439.

[28] Ranger N, Reeder T, Lowe J. Addressing ‘deep’ uncertainty over long-term climate in major infrastructure projects: four innovations of the Thames Estuary 2100 Project. EURO J Decis Process. 2013;1(3–4):233–62.

[29] Müller C. Climate Change Impacts on Sub-Saharan Africa: an overview and analysis of scenarios and models. German Development Institute; 2009. Report No.: 3/2009.

[30] Sherman M, Berrang-Ford L, Lwasa S, Ford J, Namanya DB, Llanos-Cuentas A, et al Drawing the line between adaptation and development: a systematic literature review of planned adaptation in developing countries. WIREs Clim Chang. 2016;7(7):707–26.

